# Distinct metabolic associations of subcutaneous and visceral adipocyte morphology in women with or without obesity

**DOI:** 10.1038/s41598-025-25101-5

**Published:** 2025-11-21

**Authors:** Pailin Maikaew, Trittamon Phattanakiatsakul, Chantacha Sitticharoon, Issarawan Keadkraichaiwat

**Affiliations:** Department of Physiology, Faculty of Medicine Siriraj Hospital, Mahidol University, 2 Wanglang Rd., Siriraj, Bangkoknoi, Bangkok, 10700 Thailand

**Keywords:** Adipocyte geometry, Visceral adipose tissue, Subcutaneous adipose tissue, Insulin resistance, Obesity, Biomarkers, Diseases, Endocrinology, Physiology

## Abstract

This study aimed to compare subcutaneous and visceral adipocyte geometries, clinical, metabolic, hormonal, and gene expression parameters, between participants with insulin resistance (IR; *n* = 6) and without IR (*n* = 28). Additionally, correlations between these factors and adipocyte geometries were analyzed in 27 participants, including 14 with obesity and 13 without obesity, due to limited tissue availability. In a cross-sectional study at a tertiary hospital, 34 female patients undergoing intra-abdominal surgery were recruited. Participants with IR had higher serum leptin and larger visceral adipocytes but lower serum omentin and adiponectin/leptin ratio, despite comparable gene expression. Visceral adipocyte geometries showed positive correlations with IR parameters (glucose and HOMA-IR) only in individuals without obesity, but exhibited negative correlations with QUICKI, serum adiponectin, adiponectin/leptin ratio, omentin, and/or visfatin in both groups, with generally stronger correlations observed in individuals without obesity. Importantly, enlarged adipocytes were associated with lower subcutaneous and visceral *adiponectin*, *omentin*, and *visfatin* mRNA expression in individuals with obesity, and with higher visceral LEP mRNA expression in individuals without obesity. Multiple regression analysis identified the serum adiponectin/leptin ratio as an independent predictor of adipocyte geometries in both participants with and without obesity, and body weight in individuals without obesity. In conclusion, enlarged visceral adipocytes were more strongly associated with metabolic dysregulation in individuals without obesity, while individuals with obesity exhibited mostly weaker or no correlations, possibly due to reduced capacity for further adipocyte expansion and pre-existing metabolic impairments. The serum adiponectin/leptin ratio is a sensitive biomarker reflecting adipocyte function and morphology.

**Supplementary Information:**

The online version contains supplementary material available at 10.1038/s41598-025-25101-5.

## Introduction

Obesity is a chronic and complex disease characterized by excessive fat accumulation, posing a major threat to human health and affecting quality of life^1^. It can lead to chronic low-grade systemic and local inflammation, which contributes to the development of insulin resistance (IR)^2^. The pathophysiology of IR and the mechanisms driving obesity-induced IR involve complex interactions between clinical parameters, metabolic factors, hormonal regulation, adipose tissue (including its location, lipid storage capacity, expandability, size, blood supply, and lipolytic properties), metabolic parameters, and adipokines^2^.

Adipose tissue, primarily composed of adipocytes and other cells including preadipocytes, mesenchymal stem cells, fibroblasts, vascular endothelial cells, macrophages, and various immune cells such as regulatory T cells, plays a crucial role in regulating energy storage, metabolic homeostasis, insulin sensitivity, and systemic inflammation^3,4^. Adipose tissue is categorized into 2 main types based on its location, which are subcutaneous and visceral adipose tissues, contributing to varying metabolic profiles and disease states^5^. Subcutaneous adipose tissue is located beneath the skin, distributed throughout the body, and characterized by an increased capacity to continuously store fat and expand in size^6^. In contrast, visceral adipose tissue is located around the heart (epicardial, pericardial) and intra-abdominal organs, including mesenteric, omental, retroperitoneal, perirenal, and perigonadal fat depots^7^, accumulating in confined spaces and having a lower capacity to expand in size than subcutaneous adipose tissue^8^.

In terms of adipocyte size, subcutaneous adipocytes, both in individuals with or without obesity, are larger than visceral adipocytes^9^. The blood supply to subcutaneous adipocytes drains into the systemic circulation, allowing free fatty acids (FFAs), glycerol, and other substances from lipolysis (fat breakdown) to be released into the bloodstream and distributed throughout the body^10^. In contrast, visceral adipocytes receive their blood supply from the portal circulation, facilitating the direct transfer of breakdown products to the liver, which may promote hepatic gluconeogenesis, reduce fatty acid oxidation, and increase lipid accumulation, resulting in hepatic lipotoxicity and ultimately leading to hepatic IR^10^. Furthermore, the rate of lipolysis in visceral adipocytes is higher than that in subcutaneous adipocytes due to the greater expression of the lipolytic enzyme hormone-sensitive lipase (HSL) and the lipolytic receptors β_1_- and β_2_-adrenoceptors in visceral adipocytes^11^. Conversely, subcutaneous adipocytes are more active in anti-lipolysis due to the increased activity of anti-lipolytic receptors, including insulin receptors, α₂-adrenoceptors, and adenosine receptors^12^.

Adipose tissue can produce several adipokines, which may exert specific effects on various biological processes, including insulin sensitivity (e.g., leptin, adiponectin, visfatin, and omentin); insulin secretion (e.g., apelin and nesfatin-1); inflammation (e.g., interleukin (IL)-1β, -6, -8, and -10, tumor necrosis factor (TNF), and resistin); and other metabolic functions^13^.

Although the interactions between IR and adipose tissue, including adipokines^14^, are well characterized, the specific differences in adipocyte geometries between individuals with and without IR have not been thoroughly explored. Furthermore, the correlations between adipocyte geometries and clinical, metabolic, hormonal, and gene expression parameters in subcutaneous and visceral adipose tissues in participants with or without obesity remain under-investigated. This study aimed to: (1) compare clinical, metabolic, hormonal, and gene expression parameters, and adipocyte geometries between participants with and without IR; (2) determine correlations between adipocyte geometries in subcutaneous and visceral adipose tissues and clinical, metabolic, hormonal, and gene expression parameters in participants with and without obesity; and (3) identify the factors that contribute to adipocyte geometries through multiple linear regression analyses in participants with and without obesity. These findings may provide deeper insights into the mechanisms driving obesity-related metabolic disorders, ultimately contributing to more effective interventions to reduce the risk of IR and its related complications.

## Results

### Comparisons of demographic, anthropometric, clinical, metabolic, hormonal, adipocyte geometry, and adipose tissue gene expression parameters between participants with and without obesity

Comparisons of demographic, anthropometric, clinical, metabolic, hormonal, adipocyte geometry, and adipose tissue gene expression parameters between participants with and without obesity are presented in Supplementary Table 1. These data were derived from the same cohort as previously published^9,15–19^. Minor variations or identical values in some subgroups may be observed due to differences in data inclusion and grouping across analyses. Participants with obesity had higher body weight (BW), body mass index (BMI), waist circumference (WC), hip circumference (HC), waist-to-hip ratio (WHR), serum leptin, visceral adipocyte parameters, subcutaneous adipocyte perimeter, and visceral *LEP* mRNA expression, whereas serum omentin levels were lower (*P* < 0.05 all) compared with participants without obesity (Supplementary Table 1). Serum visfatin, adiponectin, and peptide YY (PYY), and adipokine gene expression levels did not differ significantly between participants with and without obesity (Supplementary Table 1).

### Comparisons of clinical and metabolic parameters between participants with and without IR

Comparisons of clinical and metabolic parameters of participants with and without IR are shown in Table 1. In participants with IR, WC, HC, WHR, plasma glucose, plasma insulin, and the homeostatic model assessment for insulin resistance (HOMA-IR) were significantly higher (*P* < 0.05 all), with trends toward increases in BW (*P* = 0.058) and BMI (*P* = 0.052) compared to participants without IR (Table 1). On the other hand, the quantitative insulin sensitivity check index (QUICKI) was significantly lower in individuals with IR compared to those without IR (*P* < 0.001) (Table 1).

**Table 1:** Comparisons of clinical and metabolic parameters between participants with and without insulin resistance.

Factors	Participants without IR (*N* = 28)		Participants with IR (*N* = 6)		*P*-value
	Mean	SD	Mean	SD	
Age (years)	44.36	7.35	48.50	5.75	0.205
Body weight (kg)	60.49	13.65	79.55	23.08	**0.058**
Body mass index (kg/m^2^)	24.88	5.00	31.55	8.40	**0.052**
Waist circumference (cm)	81.19	10.77	101.83	17.81	*0.001*
Hip circumference (cm)	96.21	9.17	109.17	12.35	*0.006*
Waist-to-hip ratio	0.84	0.06	0.93	0.06	*0.003*
SBP (mmHg)	122.70	16.11	133.67	15.55	0.139
DBP (mmHg)	73.32	9.03	76.67	4.50	0.433
Glucose (mg/dL)	84.39	9.98	108.67	28.99	*0.011*
Insulin (µU/mL)	4.98	2.55	14.00	5.23	*0.001*
HOMA-IR	1.07	0.61	3.59	1.34	*< 0.001*
QUICKI	0.40	0.04	0.32	0.01	*< 0.001*

Bold indicates trends toward significance, whereas italics indicates significant comparisons. N = number; IR = Insulin Resistance; SD = standard deviation; SBP = systolic blood pressure; DBP = diastolic blood pressure; HOMA-IR = homeostatic model assessment for insulin resistance; QUICKI = quantitative insulin sensitivity check index.

### Comparisons of metabolic-regulating hormones between participants with and without IR

**Fig. 1:**
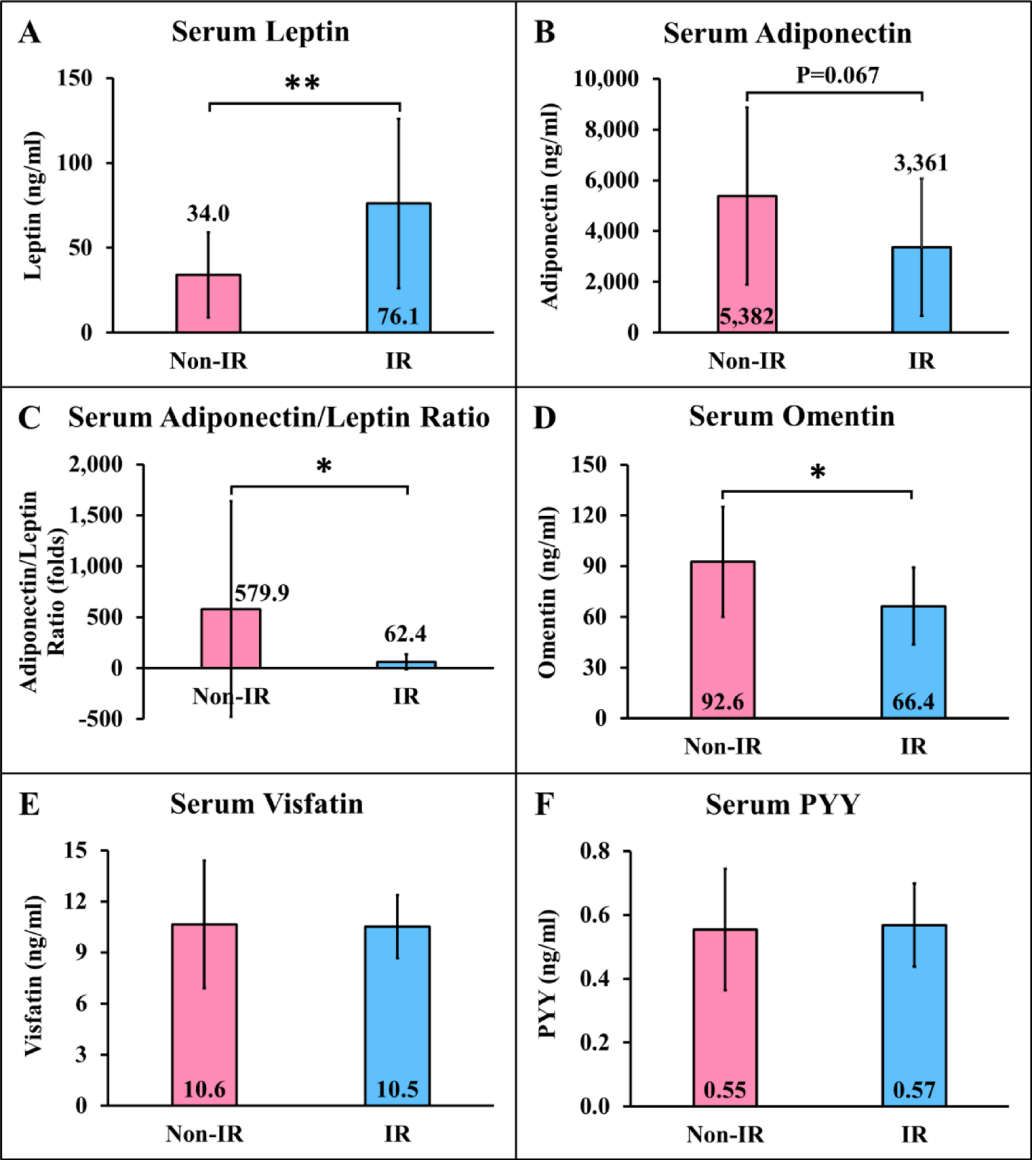
Comparisons of metabolic-regulating hormones between participants with and without insulin resistance. Non-IR = participants without insulin resistance; IR = participants with insulin resistance; PYY = peptide YY. *P < 0.05, **P < 0.01 compared between participants with and without IR.

Comparisons of metabolic-regulating hormones between participants with and without IR are shown in Fig. 1. In individuals with IR, serum leptin (Fig. 1A) was significantly higher, while the serum adiponectin/leptin ratio (Fig. 1C) and serum omentin (Fig. 1D) were significantly lower (*P* < 0.05 all), with a trend toward lower serum adiponectin (*P* = 0.067) (Fig. 1B) compared to those without IR.

### Adipocyte geometry

**Fig. 2:**
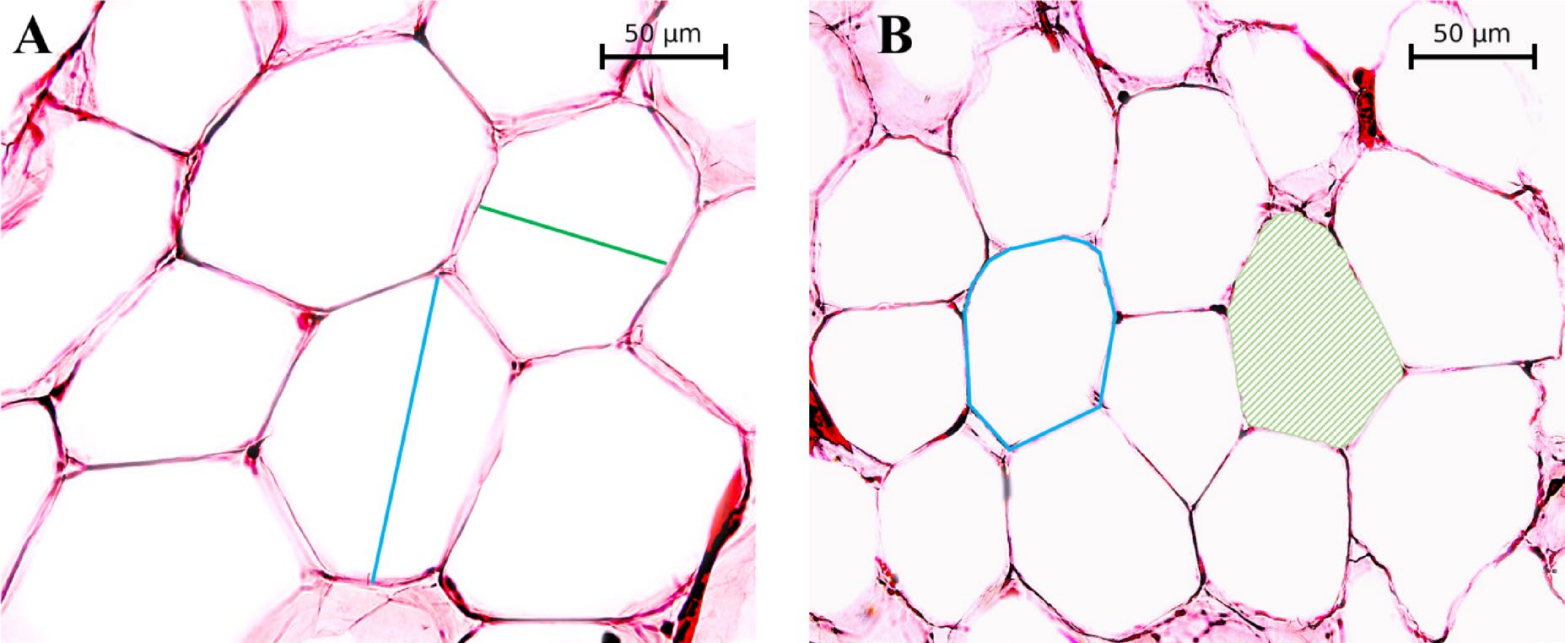
Representative histological images of subcutaneous and visceral adipose tissues. Representative histological images of adipose tissue captured with a 40× objective lens. Panel A shows subcutaneous adipose tissue and, Panel B shows visceral adipose tissue. The green line represents the shortest diameter, and the blue line indicates the longest diameter. The blue outline denotes the adipocyte perimeter, while the green shaded region represents the adipocyte area.

Representative histological images of subcutaneous (Fig. 2A) and visceral (Fig. 2B) adipose tissues are shown and were analyzed to quantify adipocyte geometries. The shortest diameter, longest diameter, perimeter, and cell area were measured to evaluate adipocyte geometries. Subcutaneous adipocytes were larger than visceral adipocytes.

### Comparisons of adipocyte geometries and gene expression between participants with and without IR

**Fig. 3:**
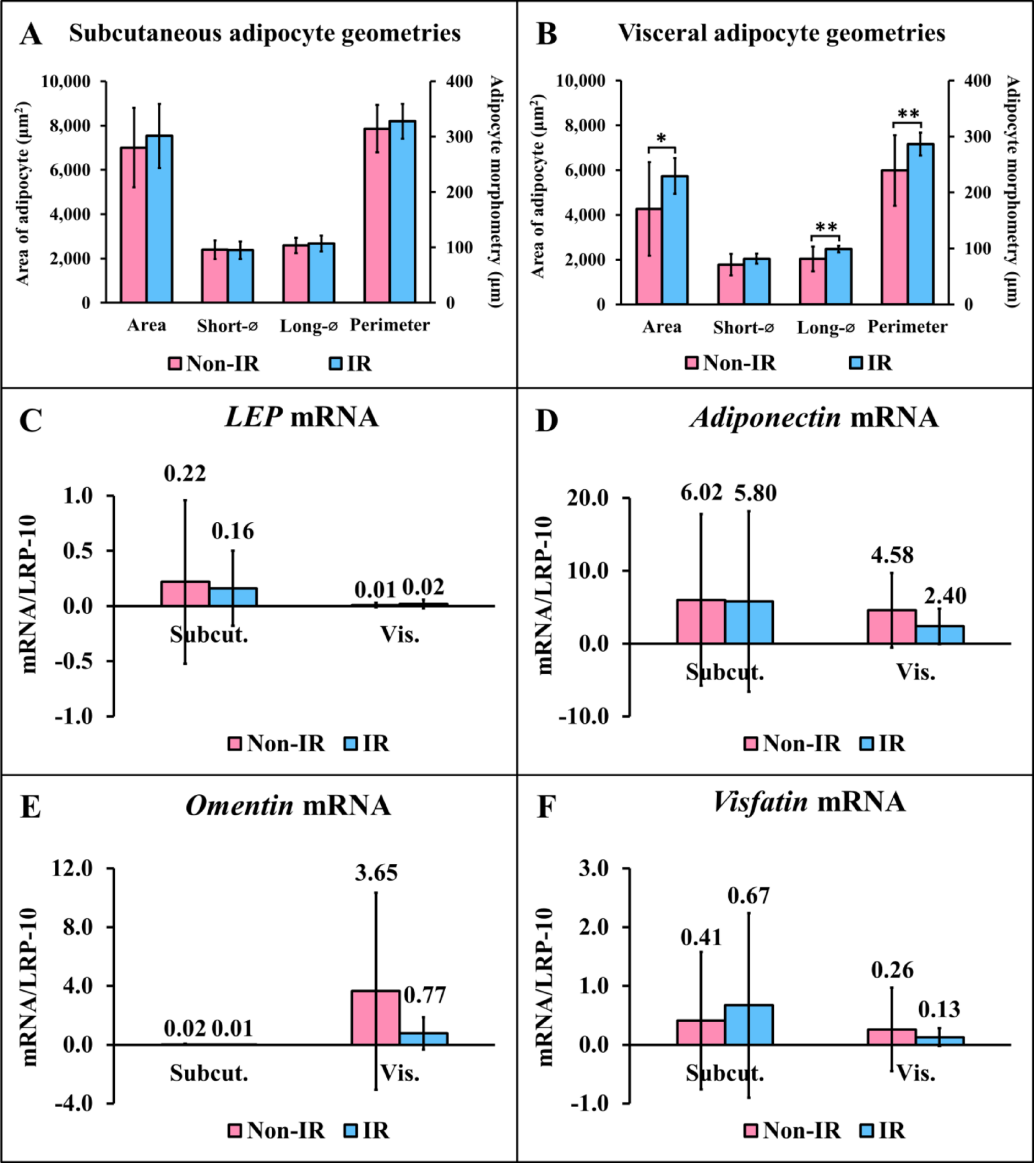
Comparisons of adipocyte geometries and gene expression between participants with and without insulin resistance. Non-IR = participants without insulin resistance; IR = participants with insulin resistance; Short-⌀ = shortest diameter; Long-⌀ = longest diameter; Subcut. = subcutaneous adipose tissue; Vis. = visceral adipose tissue. *P < 0.05, **P < 0.01 compared between participants with and without IR.

Comparisons of adipocyte geometries and gene expression between participants with and without IR are shown in Fig. 3. In individuals with IR, visceral adipocyte geometries, including area, longest diameter, and perimeter (Fig. 3B) were significantly higher compared with those without IR. *LEP* (Fig. 3C), *adiponectin* (Fig. 3D), *omentin* (Fig. 3E), and *visfatin* (Fig. 3F) mRNA expression levels were comparable between participants with and without IR in both subcutaneous and visceral adipose tissues.

### Correlations of subcutaneous and visceral adipocyte geometries with clinical, metabolic, hormonal, and gene expression parameters in participants without obesity

Correlations of subcutaneous and visceral adipocyte geometries with clinical, metabolic, hormonal, and gene expression parameters in participants without obesity are shown as a correlation heatmap in Table 2, with details provided in Supplementary Table 2. For subcutaneous adipocyte geometries, the area, longest diameter, and perimeter were positively correlated with BW; the longest diameter and perimeter with BMI and WC, while the shortest diameter, longest diameter, and perimeter were negatively correlated with serum adiponectin; and the area, shortest diameter, and perimeter with the serum adiponectin/leptin ratio (*P* < 0.05 all) (Table 2 and Supplementary Table 2).

**Table 2:**
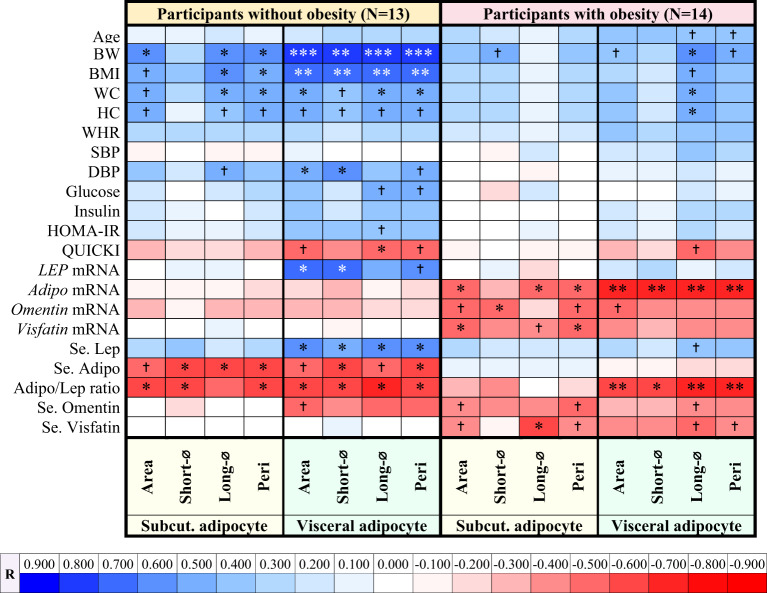
Correlation heatmap of subcutaneous and visceral adipocyte geometries with clinical, metabolic, hormonal, and gene expression parameters in participants with and without obesity.

N = number; BW = body weight; BMI = body mass index; WC = waist circumference; HC = hip circumference; WHR = waist-to-hip ratio; SBP = systolic blood pressure; DBP = diastolic blood pressure; HOMA-IR = homeostatic model assessment for insulin resistance; QUICKI = quantitative insulin sensitivity check index; Se.= serum; Adipo = adiponectin; Lep = leptin; Adipo/Lep ratio = serum adiponectin/leptin ratio; Short-⌀ = shortest diameter; Long-⌀ =longest diameter; Peri = perimeter; Subcut.= subcutaneous; ^†^ = trend toward correlation. *P < 0.05, **P < 0.01, ***P < 0.001.

For visceral adipocyte geometries, the area, shortest diameter, longest diameter, and perimeter were positively correlated with BW, BMI, and serum leptin; the area, longest diameter, and perimeter with WC, and the area and shortest diameter with diastolic blood pressure (DBP) and *LEP* mRNA expression, while the longest diameter was negatively correlated with QUICKI; the shortest diameter and perimeter with serum adiponectin; and the area, shortest diameter, longest diameter, and perimeter with the serum adiponectin/leptin ratio (*P* < 0.05 all) (Table 2 and Supplementary Table 2).

### Correlations of subcutaneous and visceral adipocyte geometries with clinical, metabolic, hormonal, and gene expression parameters in participants with obesity

Correlations of subcutaneous and visceral adipocyte geometries with clinical, metabolic, hormonal, and gene expression parameters in participants with obesity are shown as a correlation heatmap in Table 2, with details provided in Supplementary Table 2. For subcutaneous adipocytes, the area, longest diameter, and perimeter were negatively correlated with *adiponectin* mRNA expression; the shortest diameter with *omentin* mRNA expression; and the area and perimeter with *visfatin* mRNA expression; and the longest diameter with serum visfatin (*P* < 0.05 all), with trends toward negative correlations between *omentin* mRNA expression and the area and perimeter (*P* = 0.074 both) (Table 2 and Supplementary Table 2).

For visceral adipocytes, the longest diameter exhibited positive correlations with BW, WC, and HC, while the area, shortest diameter, longest diameter, and perimeter showed negative correlations with *adiponectin* mRNA expression and the serum adiponectin/leptin ratio (*P* < 0.05 all), with a trend toward a positive correlation between the longest diameter and serum leptin (*P* = 0.081) and a trend toward a negative correlation between the area and *omentin* mRNA expression (*P* = 0.088) (Table 2 and Supplementary Table 2).

### Multiple regression analysis of subcutaneous and visceral adipocyte geometries in participants with and without obesity

Multiple regression analysis of subcutaneous and visceral adipocyte geometries in participants with and without obesity is shown in Table 3. When setting the area of subcutaneous adipocytes in individuals without obesity as a dependent variable, 2 models with significant interactions were observed, using the serum adiponectin/leptin ratio (R²** =** 0.377, *P* = 0.044) (model 1) or the serum adiponectin/leptin ratio and systolic blood pressure (SBP) (R² **= **0.628, *P* = 0.019) (model 2) as independent variables (Table 3). For the perimeter of subcutaneous adipocytes in individuals without obesity as a dependent variable, a significant interaction was found, using BW (R²** =** 0.412, *P* = 0.018) as an independent variable (Table 3). By setting either the area or the perimeter of visceral adipocytes in individuals without obesity as a dependent variable, a significant interaction was found, using BW (R² = 0.686, *P* < 0.001) as an independent variable (Table 3). When setting either the area or the perimeter of visceral adipocytes in participants with obesity as a dependent variable, a significant interaction was found, using the serum adiponectin/leptin ratio (R²** =** 0.451, *P* = 0.009; R²** =** 0.525, *P* = 0.003, respectively) as an independent variable (Table 3).

**Table 3:** Multiple regression analysis of subcutaneous and visceral adipocyte geometries in participants with and without obesity.

Factor	*R*	*R* ^2^	*P*-value	Model	Coefficient	Standard error	T-value	*P*-value
Participants without obesity (*N* = 13)								
S. Area model 1	0.614	0.377	0.044	(Constant)	7,313.368	547.999	13.346	< 0.001
				Adipo/Lep ratio	− 1.080	0.463	− 2.334	0.044
S. Area model 2	0.792	0.628	0.019	(Constant)	13,913.382	2,878.663	4.833	0.001
				Adipo/Lep ratio	− 1.435	0.409	− 3.508	0.008
				SBP	− 52.378	22.565	− 2.321	0.049
S. Peri	0.642	0.412	0.018	(Constant)	111.301	68.690	1.620	0.133
				Body weight	3.682	1.327	2.775	0.018
V. Area	0.828	0.686	< 0.001	(Constant)	− 8,286.804	2,437.998	− 3.399	0.006
				Body weight	226.916	46.306	4.900	< 0.001
V. Peri	0.828	0.686	< 0.001	(Constant)	− 160.633	77.942	− 2.061	0.064
				Body weight	7.264	1.480	4.907	< 0.001
Participants with obesity (*N* = 14)								
V. Area	0.671	0.451	0.009	(Constant)	6,963.953	573.124	12.151	< 0.001
				Adipo/Lep ratio	− 13.172	4.198	− 3.138	0.009
V. Peri	0.724	0.525	0.003	(Constant)	322.454	15.292	21.086	< 0.001
				Adipo/Lep ratio	− 0.408	0.112	− 3.639	0.003

N = number; R = correlation coefficient; S. = subcutaneous; V. = visceral; Peri = perimeter; Adipo/Lep ratio = serum adiponectin/leptin ratio; SBP = systolic blood pressure.

**Fig. 4:**
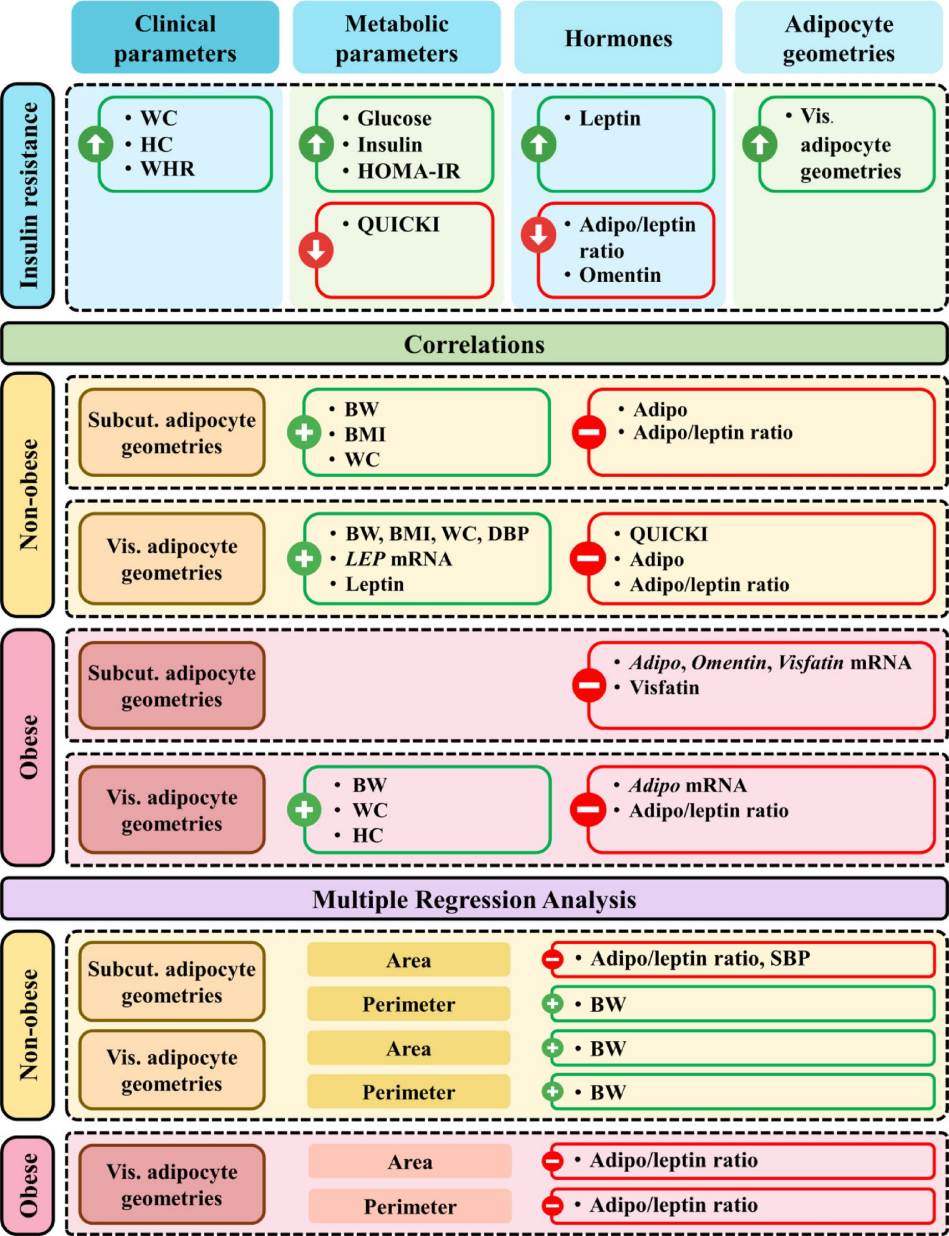
Summary of results. = higher; = lower; Non-obese = participants without obesity; Obese = participants with obesity; = positive correlations; = negative correlations; WC = waist circumference; HC = hip circumference; WHR = waist-to-hip ratio; HOMA-IR = homeostatic model assessment for insulin resistance; QUICKI = quantitative insulin sensitivity check index; Adipo/Leptin Ratio = serum adiponectin/leptin ratio; Vis.= visceral; BW = body weight; BMI = body mass index; Adipo = adiponectin; Subcut. = subcutaneous; DBP = diastolic blood pressure; SBP = systolic blood pressure.

The summary of results is shown in Fig. 4.

## Discussion

This study focused on comparisons of clinical, metabolic, hormonal, adipocyte geometry and gene expression parameters between participants with and without IR and on examining correlations of adipocyte geometries with these factors in participants with or without obesity. To the best of our knowledge, this is the first study to compare adipocyte geometries between individuals with and without IR. Additionally, it is the first to determine the correlations of adipocyte geometries with clinical parameters, insulin sensitivity parameters, and adipokines, analyzed separately in participants with or without obesity.

**Fig. 5:**
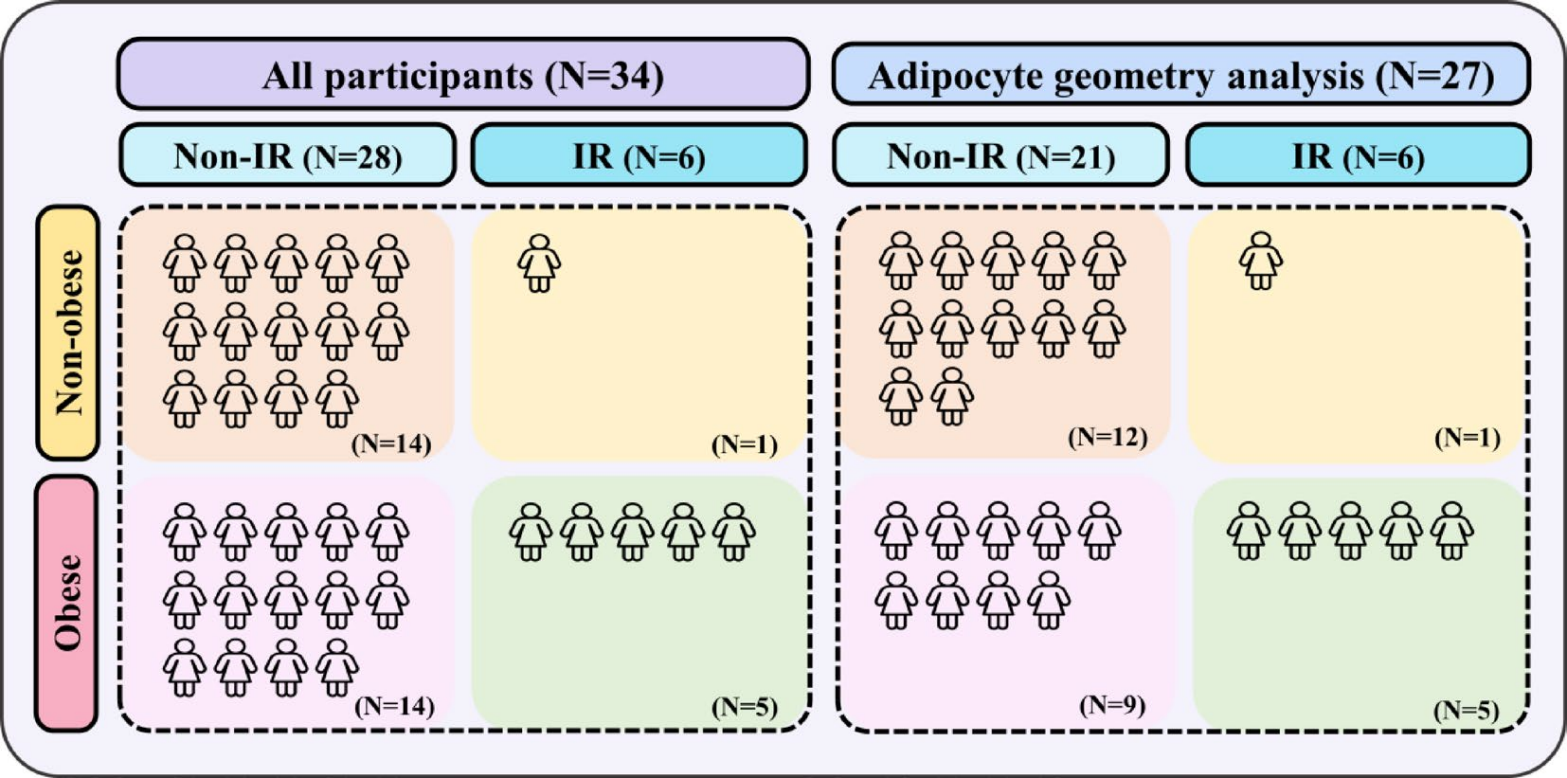
Allocation of participants. N = number; Non-IR = participants without insulin resistance; IR = participants with insulin resistance. Non-obese = participants without obesity; Obese = participants with obesity.

In all participants, 82.35% (28/34) were participants without IR, and 17.65% (6/34) were participants with IR. Among participants without IR, 50% (14/28) did not have obesity and 50% (14/28) had obesity, while in participants with IR, 16.67% (1/6) did not have obesity and 83.33% (5/6) had obesity (Fig. 5). Conversely, participants without obesity, 93.33% (14/15) were participants without IR and 6.67% (1/15) were IR, whereas in participants with obesity, 73.68% (14/19) were participants without IR and 26.32% (5/19) were IR (Fig. 5). These results indicate that obesity is a significant risk factor for the development of IR; however, the presence of IR is not exclusively dependent on obesity, as 6.67% of participants without obesity had IR (Fig. 5).

This study showed that individuals with IR exhibited increased IR-related parameters, including plasma glucose and insulin levels, and HOMA-IR, but reduced insulin sensitivity-related parameters, including QUICKI, serum adiponectin, omentin, and adiponectin/leptin ratio. Furthermore, participants with IR showed increased visceral adiposity, but not subcutaneous adiposity, along with higher WC, HC, and WHR, as well as elevated serum leptin levels and a trend toward higher BW and BMI, suggesting that visceral adiposity, rather than subcutaneous adiposity, is associated with IR. This might be explained by the fact that visceral adipose tissue, confined to the abdominal cavity, releases higher amounts of FFAs, which can lead to hepatic lipotoxicity and IR^10^. In contrast, subcutaneous fat expands more easily and releases FFAs more slowly, thereby reducing its impact on liver function and IR^10^.

Despite comparable *LEP*, *adiponectin*, and *omentin* gene expression between participants with and without IR in subcutaneous and visceral adipose tissues, serum leptin levels were higher whereas serum omentin levels were lower, with a trend toward lower serum adiponectin in participants with IR compared to participants without IR. The dissociation between *LEP* mRNA and circulating leptin supports previous findings that insulin, a robust stimulator of leptin secretion^20^, can enhance its release from adipose tissues without altering leptin transcription^21^, which likely underlies the higher circulating leptin levels associated with IR. In contrast, the lower serum adiponectin and omentin levels in participants with IR, despite comparable gene expression, are likely attributable to the chronic low-grade inflammation that characterizes IR. Elevated pro-inflammatory cytokines such as TNF-α, IL-1β, and interferon-γ (IFN-γ)^22^ have been shown to suppress adiponectin secretion from adipocytes^23^, while omentin secretion was reduced by insulin and glucose in human omental adipose tissue and in inflammatory conditions, including polycystic ovary syndrome^24^ and inflammatory bowel disease^25^. Notably, serum visfatin levels were comparable between participants with and without IR, indicating that IR is unlikely to be a major determinant of visfatin concentrations, which is consistent with a previous finding that HOMA-IR is not associated with serum visfatin levels^26^.

Age tended to be positively correlated with visceral adipocytes only in participants with obesity, showing a moderate correlation coefficient (*R* = 0.41–0.50), but not in participants without obesity (Table 2 and Supplementary Table 2). This suggests that, in individuals with obesity, increasing age is associated with larger visceral adipocytes, which is consistent with a previous study^27^. Aging alters adipose tissue dynamics and distribution^28^, favoring hypertrophy over hyperplasia and reducing adipogenesis^8^, leading to excess lipid storage, particularly in visceral fat^29^. Subcutaneous adipose tissue, unlike visceral fat, does not exhibit a significant positive correlation with age and its expansion is not significantly influenced by the aging-related decline in adipogenesis^27^.

Adipocyte geometries exhibited positive correlations with BW, BMI, and WC, and trends toward positive correlations with HC in both subcutaneous and visceral adipocytes in individuals without obesity; and positive correlations with BW, WC, and HC in visceral adipocytes, along with a trend toward a positive correlation with BW in subcutaneous adipocytes and with BMI in visceral adipocytes, with a lower correlation coefficient observed in participants with obesity (Table 2 and Supplementary Table 2). In multiple linear regression analysis, BW was the major contributor to subcutaneous and visceral adipocyte geometries only in individuals without obesity. These findings suggest that, in individuals without obesity, changes in adipocyte size and shape are more closely linked to general body composition and fat distribution than in individuals with obesity. In participants without obesity, there is more space available for both subcutaneous and visceral fat cells to expand, allowing adipocyte geometry to more closely mirror changes in BW, BMI, and WC. In contrast, the weaker correlations in individuals with obesity suggest a more complex, possibly dysregulated relationship between adipocyte geometry and body composition, especially in the subcutaneous fat compartment. As adipocytes in individuals with obesity have already expanded to their limits, their geometry may no longer respond as sensitively to changes in body size or fat distribution.

WC exhibited positive correlations with visceral adipocyte geometries in both participants with and without obesity but with subcutaneous adipocyte geometries in only participants without obesity (Table 2 and Supplementary Table 2). This suggests that visceral adiposity may be a more consistent determinant of central fat accumulation across different levels of adiposity, while subcutaneous fat contribution to WC becomes less prominent in obesity, possibly due to reaching a threshold beyond which further expansion does not proportionally reflect changes in WC. Visceral, but not subcutaneous, adipocyte geometries exhibited trends toward positive correlations with plasma glucose and HOMA-IR, and showed a significant negative correlation with QUICKI in individuals without obesity, as well as a trend toward a negative correlation with QUICKI in individuals with obesity (Table 2 and Supplementary Table 2). Expansion of visceral adipocytes in the confined space causes FFAs to enter the portal circulation directly, leading to fat accumulation in the liver, which results in hepatic lipotoxicity and increased gluconeogenesis, thereby inducing IR^10^. In contrast, subcutaneous adipocytes, being distributed over a larger area, contribute to less FFA toxicity and lower IR^10^. The stronger correlation observed in participants without obesity may be due to the lower overall level of adiposity, which allows for more adipocyte expansion, making visceral adipocyte geometries more directly related to IR parameters; whereas in participants with obesity, limited capacity for further expansion may underlie the weaker associations.

DBP exhibited significant positive correlations with visceral adipocyte geometries and a trend toward a positive correlation with subcutaneous adipocyte geometry only in participants without obesity, but not in participants with obesity (Table 2 and Supplementary Table 2). These findings suggest that, in individuals without obesity, increasing adipocyte size, particularly in visceral fat, may contribute to elevated DBP. As adipocyte size increases, it leads to greater triglyceride accumulation, resulting in local hypoxia within adipose tissue. This hypoxic state induces the expression of pro-inflammatory cytokines such as TNF-α, IL-1, and IL-6^30^, promoting chronic inflammation. The inflammatory environment contributes to arterial stiffness and endothelial dysfunction, primarily through reduced nitric oxide (NO) bioavailability, ultimately leading to vasoconstriction and increased vascular resistance^31^, which in turn elevates DBP. Such correlations were observed only in individuals without obesity, possibly because adipose tissue expansion still plays a dynamic role in modulating vascular function in this population. The stronger correlation with visceral adipocyte size may be explained by the fact that visceral fat is more metabolically active and more prone to inflammation and cytokine release than subcutaneous fat, leading to a greater impact on vascular tone and resistance. In contrast, the lack of association in individuals with obesity may reflect that vascular changes are already established and less responsive to further adipocyte enlargement.

Visceral adipocyte geometries exhibited significant positive correlations with serum leptin levels and *LEP* mRNA expression in participants without obesity, as well as a trend toward a positive correlation with serum leptin levels in participants with obesity (Table 2 and Supplementary Table 2). However, no correlation was observed between subcutaneous adipocyte geometries and serum leptin levels or *LEP* mRNA expression in participants with and without obesity. This suggests that *LEP* expression and leptin production/secretion may be more closely linked to visceral, rather than subcutaneous adipocyte size. Although *LEP* expression^32,33^ and leptin secretion rate^34^ are significantly higher in subcutaneous than in visceral adipose tissues, subcutaneous adipocytes may already exhibit high baseline leptin output and show limited further increases with hypertrophy. In contrast, visceral adipocytes, which start from a lower baseline, may have greater capacity to upregulate *LEP* expression and/or leptin production/secretion as cell size increases, resulting in a stronger correlation. In individuals with obesity, the weaker correlation of serum leptin levels might stem from the fact that leptin secretion from adipose tissue was higher compared to individuals without obesity^34^, allowing for less additional secretion in response to further adipocyte enlargement, and thus attenuating the association between leptin levels and adipocyte geometries.

Both subcutaneous and visceral adipocyte geometries exhibited significant negative correlations with *adiponectin* mRNA expression only in participants with obesity, while with serum adiponectin levels only in participants without obesity (Table 2 and Supplementary Table 2). These findings suggest that larger subcutaneous and visceral adipocyte sizes are associated with lower *adiponectin* mRNA expression in individuals with obesity but lower serum adiponectin levels in individuals without obesity. A previous study showed that enlarged adipocytes lead to an inflammatory state, which reduces adiponectin expression^35^and secretion^23^. In individuals with obesity, higher BMI is associated with greater inflammation, including increased serum levels of TNF, IL-6, and C-reactive protein (CRP)^36^. This inflammatory response may further suppress *adiponectin* gene expression, thereby explaining the significant negative correlations observed in this group. However, serum adiponectin did not correlate with either subcutaneous or visceral adipocyte geometries in participants with obesity, indicating that adiponectin levels in individuals with obesity are not directly proportional to adipocyte size. This is consistent with a previous study showing that serum adiponectin negatively correlated with visceral fat area in those with < 100 cm², but not in those with ≥ 100 cm², due to low variability^37^. Since obesity is associated with a chronic low-grade inflammatory state^38^, this progressively impairs adiponectin secretion over time^39^, ultimately resulting in reduced adiponectin levels regardless of adipocyte size^37^.

The serum adiponectin/leptin ratio exhibited significant negative correlations with both subcutaneous and visceral adipocyte geometries in participants without obesity and with visceral adipocyte geometries in participants with obesity (Table 2 and Supplementary Table 2). Compared with adiponectin alone, the serum adiponectin/leptin ratio showed a higher correlation coefficient in individuals without obesity and reached statistical significance in visceral adipocytes of individuals with obesity (Table 2 and Supplementary Table 2). This may be because dividing by leptin helps better reflect the secretory function of adipocytes in terms of leptin output. Thus, the serum adiponectin/leptin ratio may provide a more accurate and sensitive biomarker for assessing both adipocyte secretory function and the overall morphological status of adipose tissue, particularly under metabolic stress such as obesity. This is further supported by a previous study showing that the serum adiponectin/leptin ratio serves as a reliable marker of adipose tissue dysfunction and may effectively estimate cardiometabolic risk linked to obesity and metabolic syndrome, enabling the identification of a greater number of at-risk individuals^40^.

*Omentin* mRNA expression exhibited a significant negative correlation with subcutaneous adipocyte geometry and a trend toward a negative correlation with visceral adipocyte geometry in individuals with obesity (Table 2 and Supplementary Table 2). Serum omentin levels exhibited trends toward negative correlations in both subcutaneous and visceral adipocyte geometries in participants with obesity, and with visceral adipocyte geometry in those without obesity (Table 2 and Supplementary Table 2). These findings suggest that adipocyte enlargement may impair omentin expression and secretion in both subcutaneous and visceral adipose tissues in individuals with obesity, but only omentin secretion from visceral adipose tissue in individuals without obesity. This interpretation is consistent with previous reports showing that *omentin* expression is reduced in visceral adipose tissue^41^, and circulating levels^16,41^ in individuals with obesity. However, in participants without obesity, only visceral adipocytes, which are confined to a limited space and serve as the primary site of omentin synthesis, are primarily affected^16^, since *omentin* gene expression in subcutaneous adipose tissue is much lower than in visceral adipose tissue^42^.

Subcutaneous *visfatin* gene expression exhibited negative correlations with subcutaneous adipocyte geometries while serum visfatin levels showed negative correlation with subcutaneous adipocyte geometries and trends toward negative correlations with visceral adipocyte geometries only in participants with obesity (Table 2 and Supplementary Table 2). These results suggest that in individuals with obesity, enlargement of subcutaneous adipocytes is associated with decreased *visfatin* expression, which is consistent with a previous study demonstrating that adipocyte hypertrophy in obesity is associated with decreased *visfatin* expression in subcutaneous adipose tissue^42^. The negative correlations of subcutaneous and visceral adipocyte enlargement with serum visfatin levels observed only in individuals with obesity suggests that adipocyte hypertrophy in obesity may contribute to altered visfatin secretion. Adipocyte enlargement is known to be associated with local hypoxia, macrophage infiltration, and pro-inflammatory cytokine production, which can impair adipokine expression and release^43^. In individuals without obesity, adipocyte geometries were not correlated with visfatin expression or circulating levels, which is consistent with previous studies showing no correlation between adiposity and visfatin expression or serum concentrations in combined participants with and without obesity^44^.

## Limitations

This study has several limitations. First, the menstrual cycle phase of female participants was not controlled due to irregular menstruation, primarily caused by uterine myomas. Second, the unequal distribution of participants with and without IR may have introduced statistical bias and affected the reliability of group comparisons, potentially limiting the generalizability of the findings. Third, the relatively small sample sizes for participants with and without obesity may have reduced the statistical power to detect significant correlations. Fourth, missing data in some variables may have limited the scope of certain analyses; among the 13 participants without obesity, only 12 had serum leptin and adiponectin data, and 11 had serum omentin and visfatin data, while among the 14 participants with obesity, only 13 had available SBP and DBP data. Fifth, protein expression in adipose tissues could not be measured due to the limited amount of tissue available.

## Conclusion

This study investigated clinical, metabolic, hormonal, adipocyte geometry, and gene expression profiles in comparisons between participants with and without IR, and examined correlations of adipocyte geometries with these factors in individuals with and without obesity. When stratified by IR, participants with IR exhibited larger visceral adipocytes and higher serum leptin levels but lower serum omentin and adiponectin/leptin ratio compared with participants without IR, despite comparable adipokine gene expression. Visceral adipocyte geometries showed positive correlations with IR parameters (glucose and HOMA-IR) only in individuals without obesity, but exhibited negative correlations with QUICKI, serum adiponectin, adiponectin/leptin ratio, omentin, and/or visfatin in both groups, with generally stronger correlations observed in individuals without obesity. These findings suggest that visceral adipocyte hypertrophy plays a key role in the pathophysiology of IR and cardiovascular risk, probably through pro-inflammatory signaling and endocrine dysfunction. In contrast, individuals with obesity exhibited mostly weaker or no correlations, possibly due to reduced capacity for further adipocyte expansion and pre-existing vascular or metabolic impairments. Importantly, adipocyte hypertrophy was associated with lower *adiponectin*, *omentin*, and *visfatin* mRNA expression in both subcutaneous and visceral adipose tissues in individuals with obesity, and with higher visceral *LEP* mRNA expression in individuals without obesity. Multiple regression analysis identified the adiponectin/leptin ratio as an independent predictor of adipocyte geometries in both participants with and without obesity, and BW in participants without obesity. The serum adiponectin/leptin ratio is a sensitive biomarker reflecting adipocyte function and morphology. Overall, this study highlights the clinical relevance of assessing adipocyte morphology, distribution, and gene expression, particularly in visceral fat, as a potential indicator of metabolic health and as targets for future therapeutic interventions aimed at preventing or mitigating IR and its complications.

## Materials and methods

### Subjects

The Siriraj Institutional Review Board at the Faculty of Medicine Siriraj Hospital, Mahidol University, Thailand, approved the study protocols (COA No. Si 533/2009, Si 490/2011, and Si 423/2013) in compliance with international guidelines for human research protection, including the Declaration of Helsinki, the Belmont Report, the CIOMS Guidelines, and the International Conference on Harmonization in Good Clinical Practice (ICH-GCP). Informed consent was obtained from all 34 participants.

Inclusion criteria were patients undergoing intra-abdominal surgery, while exclusion criteria encompassed those on endocrine therapy (e.g., hormone replacement therapy, steroids, thyroxine), undergoing endocrine-related operations, traumatic operations, severe abdominal inflammation, malignancy, pregnancy, or lactation.

In this study, only female participants were recruited because male patients undergoing open abdominal surgery mainly had cancer or emergency conditions, which fell under the exclusion criteria. Most of the female participants had irregular menstruation due to myoma uteri, so menstrual cycle phases could not be controlled.

Participants were categorized by IR using a HOMA-IR threshold of > 2.3^45^. In participants without IR, SBP and DBP data for 1 participant were missing due to unavailability in the case record form. Serum adiponectin, omentin, and visfatin data for 2 participants without IR and serum leptin data for 1 participant with IR were missing due to insufficient blood samples for measurement.

Participants were also categorized by BMI as without obesity (*N* = 15; BMI < 25) and with obesity (*N* = 19; BMI ≥ 25), based on the criteria for the Asian population^46^. The allocation of participants is shown in Fig. 5.

### Sample size calculation

Participants of this study were obtained from the same cohort from our previous study^15^, which calculated sample size from correlation analysis between subcutaneous and visceral adipose tissue *neuropeptide Y* (*NPY*) and *NPY receptor* mRNA expression and clinical, anthropometric, and metabolic parameters to detect a correlation coefficient of *r* = 0.5 with a type I error (α) of 0.05 and a type II error (β) of 0.20 (power = 80%), which indicated that approximately 30 participants were needed. Accordingly, 34 participants were recruited to account for potential dropouts, under the approved study protocols (Siriraj Institutional Review Board COA No. Si 533/2009)^15^. That publication included only participants with normal weight and participants with obesity^15^, whereas participants with underweight and overweight were excluded. In the present study, all 34 participants were analyzed, including those with underweight and overweight classified as participants without obesity, resulting in 15 participants without obesity and 19 participants with obesity.

Subsequently, this cohort was further analyzed to compare clinical, metabolic, hormonal, adipocyte geometry, and gene expression parameters between participants with and without IR, as clinically meaningful. However, the small number of participants with IR was insufficient to allow meaningful correlation analyses. Given the importance of adipocyte geometries in different metabolic states, we therefore performed correlation analyses in participants with and without obesity, where the larger sample sizes enabled more reliable evaluation.

### Demographic, anthropometric, and clinical data of participants

Demographic, anthropometric, and clinical data of participants, including age, BW, BMI, WC, HC, WHR, SBP, and DBP were obtained from medical records.

### Tissue and blood collection

Blood was collected in the fasting state prior to the operation. Four to five pieces of abdominal subcutaneous and omental (visceral) adipose tissue, each measuring 0.5 cm^3^, were obtained from each patient during the operation. The adipose tissues were snap-frozen in liquid nitrogen immediately and stored at -80 °C until analysis.

### Glucose and insulin assays

Plasma glucose and insulin were measured by the central laboratory of the Department of Clinical Pathology, Faculty of Medicine Siriraj Hospital, Mahidol University, Thailand. Plasma glucose was analyzed using an immunoturbidimetric assay (Hitachi, Chiyoda, Tokyo, Japan), and plasma insulin was measured using an electrochemiluminescence immunoassay (Lincoln, Madera County, CA, USA). Fasting plasma glucose and insulin levels were used to assess IR via the HOMA-IR method and insulin sensitivity via the QUICKI method. HOMA-IR was calculated as fasting glucose (mg/dL) multiplied by fasting insulin (µU/mL) divided by 405^47^ and QUICKI was calculated as the inverse of the sum of the logarithms of fasting insulin (µU/mL) and fasting glucose (mg/dL)^47^.

### Analysis of serum leptin, adiponectin, omentin, visfatin, and PYY levels

Fasting blood samples were weight-balanced and separated by centrifugation at 5,000 rpm, 4 °C for 15 min. Serum samples were stored at -80 °C until analysis. Serum leptin (Cat. No. EK-003-12; Phoenix Pharmaceuticals Inc., Burlingame, CA, USA), adiponectin (Cat. No. EK-ADI-01; Phoenix Pharmaceuticals Inc., Burlingame, CA, USA), and omentin (Cat. No. EZH0MNTN1-29 K; EMD Millipore Corporation, St. Charles, MO, USA) were measured using commercial enzyme-linked immunosorbent assay (ELISA) kits. Serum visfatin (Cat. No. EK-003-80) and PYY (Cat. No. EK-059-02) levels were analyzed using enzyme immunoassay (EIA) kits (Phoenix Pharmaceuticals Inc., Burlingame, CA, USA). The detection ranges and the minimum detectable concentrations were 0.31-20 ng/ml and 0.312 ng/ml, respectively for serum leptin, 0.15-10 ng/ml and 0.15 ng/ml, respectively for serum adiponectin, 2-200 ng/ml and 0.23 ng/ml, respectively for serum omentin, 0.1-1,000 ng/ml and 1.85 ng/ml, respectively for serum visfatin, and 1-100 ng/ml and 0.06 ng/ml, respectively for serum PYY. The absorbances were read at 450 nm using a Synergy HT Multi-Detection Microplate Reader (BioTek Instruments, Inc., Winooski, VT, USA).

### Adipocyte geometry

Subcutaneous and visceral adipocyte geometries were examined in 27 participants (13 participants without obesity and 14 participants with obesity) as shown in Fig. 5, since some samples were used for gene expression studies, leaving insufficient tissue for geometry analysis. Adipose tissue was embedded in paraffin and sectioned at 20 μm using a microtome (Global Medical Instrumentation Inc, Ramsey, MN, USA). The sections were mounted on hydrophilic Twin-Mark microscope slides (Citotest Labware Manufacturing, Nanjing, China), with the top of the slides covered by opaque tape to blind subject identification. Histological staining was performed using hematoxylin and eosin (H&E) to assess adipocyte characteristics. Adipocyte geometry was analyzed by a single observer using AxioVision^®^ software Release 4.8.2 (Carl Zeiss AG, Oberkochen, Germany), measuring the area, shortest diameter, longest diameter, and perimeter of adipocytes. For each slide, 20 random locations were selected and analyzed, and the same areas were used for all slides.

### Gene expression

Gene expression of *LEP*, *adiponectin*, *omentin*, and *visfatin* was analyzed by real-time polymerase chain reaction (PCR) as previously published^16,19^. Briefly, RNA was isolated using TRIzol^®^ reagent (Invitrogen, Carlsbad, CA, USA) according to the manufacturer’s protocol. *Low density lipoprotein related protein 10* (*LRP10*) was selected as the reference gene because it is the most stably expressed in adipose tissue. The primers for *LEP*,* adiponectin*, *omentin*, *visfatin*, and *LRP10* were the same as those reported in our previous publications^16,19^ as follows:

- *LEP*-Forward^19^ 5’-CAATGACATTTCACACACGCAGTC-3’.
- *LEP*-Reverse^19^ 5’-GCCACCACCTCTGTGGAGTAG-3’.
- *Adiponectin*-Forward^16^ 5’-GCCTCTTCAAGAAGGACAAGGC-3’.
- *Adiponectin*-Reverse^16^ 5’-CCCCATACACCTGGAGCCAG-3’.
- *Visfatin*-Forward^16^ 5’-AAGAGACTGCTGGCATAGGA-3’.
- *Visfatin*-Reverse^16^ 5’-ATGGTACTGTGTTCTGCTGC-3’.
- *Omentin*-Forward^16^ 5’-AACAGCTCCCTGCTGAGGTA-3’.
- *Omentin*-Reverse^16^ 5’-TCATAGACCACAGGGATCAC-3’.
- *LRP-10*-Forward^16,19^ 5’-GATGGAGGCTGAGATTGTGCA-3’.
- *LRP-10*-Reverse^16,19^ 5’-TGGAGTCATATCCTGGCGTAAG-3’.

All primers were designed to span exon-exon junctions, and their specificity for the target genes was confirmed using BLAST analysis. The amplicon sizes of *LEP*, *adiponectin*, *omentin*, *visfatin*, and *LRP10* were 312 base pairs (bp), 132 bp, 149 bp, 148 bp, and 169 bp, respectively. VeriQuest SYBR Green qPCR Master Mix (Affymetrix, Santa Clara, CA, USA) was used for the real-time PCR analysis. The annealing temperature was 59 °C for all genes, except *LEP* (58 °C). No template control (NTC) was included in every real-time PCR reaction as a negative control. The size of the real-time PCR products was confirmed by gel electrophoresis. Gene expression was quantified using the 2^−ΔCT^ method.

### Statistics

Statistical analysis was performed using the Statistical Package for the Social Sciences (SPSS) version 30. Data are presented as mean ± standard deviation (SD). Normality was assessed using the Kolmogorov-Smirnov test. Comparisons between participants with and without IR were conducted using the independent samples t-test for normally distributed data or a nonparametric test for non-normally distributed data. Correlation coefficients were calculated using two-tailed Pearson’s product-moment correlation for normally distributed variables or Spearman’s rank-order correlation for non-normally distributed variables. Correlations between adipocyte geometries and gene expression in adipose tissue were determined only within the same adipose tissue type but not across different depots. Multiple linear regression analysis was performed using the stepwise method to identify predictors of subcutaneous and visceral adipocyte geometries, focusing on area and perimeter as representative measures of overall cell size. In each model, only the most appropriate independent variable among highly interrelated factors (e.g., BMI, BW, and WC; or QUICKI, HOMA-IR, and insulin) was included to minimize multicollinearity. A p-value < 0.05 was considered statistically significant.

## Supplementary Information

Below is the link to the electronic supplementary material.

Supplementary Material 1

## Data Availability

The data that support the findings of this study are available from the corresponding author, upon reasonable request.
