# Supplementary material for: Distinct metabolic associations of subcutaneous and visceral adipocyte morphology in women with or without obesity
Source: Sci Rep. 2025 Nov 21;15:41287. doi: 10.1038/s41598-025-25101-5 (PMC12639111; doi:10.1038/s41598-025-25101-5)
Supplement: Supplementary file 1 — Supplementary Material 1 [file 41598_2025_25101_MOESM1_ESM.docx]

**Supplementary**

**Supplementary Table 1** Demographic, anthropometric, clinical, metabolic, hormonal, adipocyte geometry, and adipose tissue gene expression parameters stratified by obesity status

These data were derived from the same cohort as previously published^9,15-19^. Some values may be identical, while others differ slightly due to subgrouping and data inclusion criteria.

| **Factors** | **Participants without obesity** | | **Participants**  **with obesity** | | **P-value** | **Reference(s) (previous report[s])** |
| --- | --- | --- | --- | --- | --- | --- |
|  | **Mean** | **SD** | **Mean** | **SD** |  |  |
| Age (years) | 42.67 | 7.12 | 46.75 | 6.75 | 0.093 | ^15, 16^ |
| Body weight (kg) | 50.75 | 7.21 | 74.50 | 14.83 | <0.001 | ^16, 17^ |
| Body mass index (kg/m^2^) | 20.87 | 2.22 | 30.21 | 4.91 | <0.001 | ^15, 16^ |
| Waist Circumference (cm) | 74.46 | 7.48 | 93.07 | 12.88 | <0.001 | ^16^ |
| Hip Circumference (cm) | 89.86 | 6.00 | 105.60 | 8.55 | <0.001 | ^16^ |
| Waist-to-hip ratio | 0.83 | 0.05 | 0.88 | 0.07 | 0.020 | ^16^ |
| SBP (mmHg) | 119.93 | 14.77 | 128.74 | 16.45 | 0.115 | ^15^ |
| DBP (mmHg) | 72.20 | 8.78 | 76.63 | 8.37 | 0.143 | ^15^ |
| Glucose (mg/dL) | 85.60 | 11.58 | 91.11 | 20.64 | 0.363 | ^16^ |
| Insulin (μU/mL) | 5.26 | 3.41 | 7.60 | 5.30 | 0.148 | ^16^ |
| HOMA-IR | 1.18 | 0.83 | 1.78 | 1.44 | 0.158 | ^16^ |
| QUICKI | 0.40 | 0.06 | 0.37 | 0.04 | 0.066 | ^16^ |
| Serum Leptin (ng/mL) | 17.35 | 13.21 | 57.41 | 31.84 | <0.001 | ^19^ |
| Serum Adiponectin (ng/mL) | 6,130.18 | 3,864.81 | 4,126.04 | 2,818.92 | 0.100 | ^16^ |
| Serum Omentin (ng/mL) | 103.81 | 30.88 | 75.48 | 28.70 | 0.011 | ^16^ |
| Serum Visfatin (ng/mL) | 10.56 | 3.38 | 10.58 | 3.54 | 0.989 | ^16^ |
| Serum PYY (ng/mL) | 0.55 | 0.16 | 0.60 | 0.19 | 0.436 | ^18^ |
| Subcut. Area | 6,449.99 | 1,750.04 | 7,696.44 | 1,505.16 | 0.053 | ^9^ |
| Subcut. Shortest Diameter | 89.51 | 15.56 | 101.29 | 15.48 | 0.056 | ^9^ |
| Subcut. Longest Diameter | 99.07 | 13.25 | 108.53 | 12.97 | 0.068 | ^9^ |
| Subcut. Perimeter | 300.12 | 42.08 | 332.17 | 33.60 | 0.034 | ^9^ |
| Vis. Area | 3,577.42 | 1,765.17 | 5,535.27 | 1,688.58 | 0.007 | ^9^ |
| Vis. Shortest Diameter | 64.31 | 15.91 | 82.23 | 15.44 | 0.007 | ^9^ |
| Vis. Longest Diameter | 75.05 | 21.19 | 94.53 | 16.34 | 0.013 | ^9^ |
| Vis. Perimeter | 219.15 | 56.48 | 278.25 | 48.43 | 0.007 | ^9^ |
| Subcut. *LEP* mRNA | 0.33 | 0.98 | 0.11 | 0.23 | 0.394 | - |
| Vis. *LEP* mRNA | 0.00 | 0.01 | 0.02 | 0.03 | 0.044 | - |
| Subcut. *Adiponectin* mRNA | 7.36 | 15.59 | 4.93 | 8.06 | 0.597 | ^16^ |
| Vis. *Adiponectin* mRNA | 3.44 | 5.14 | 4.62 | 4.52 | 0.528 | ^16^ |
| Subcut. *Omentin* mRNA | 0.03 | 0.05 | 0.01 | 0.01 | 0.187 | ^16^ |
| Vis. *Omentin* mRNA | 1.38 | 2.09 | 4.32 | 7.73 | 0.214 | ^16^ |
| Subcut. *Visfatin* mRNA | 0.59 | 1.51 | 0.35 | 0.98 | 0.619 | ^16^ |
| Vis. *Visfatin* mRNA | 0.08 | 0.12 | 0.35 | 0.82 | 0.274 | ^16^ |

Data in this table were obtained in the present study. References indicate variables that have been previously reported from the same cohort.

SD=standard deviation; SBP=systolic blood pressure; DBP=diastolic blood pressure; HOMA-IR=homeostatic model assessment for insulin resistance; QUICKI=quantitative insulin sensitivity check index; PYY=peptide YY; Subcut.=subcutaneous; Vis.=visceral.

**Supplementary Table 2** Correlations of subcutaneous and visceral adipocyte geometries with clinical, metabolic, hormonal, and gene expression parameters in participants with and without obesity

| **Factors** | **Subcutaneous adipocyte geometries** | | | | | | | | **Visceral adipocyte geometries** | | | | | | | |
| --- | --- | --- | --- | --- | --- | --- | --- | --- | --- | --- | --- | --- | --- | --- | --- | --- |
|  | **Area** | | **Short-⌀** | | **Long-⌀** | | **Perimeter** | | **Area** | | **Short-⌀** | | **Long-⌀** | | **Perimeter** | |
|  | **R** | **P** | **R** | **P** | **R** | **P** | **R** | **P** | **R** | **P** | **R** | **P** | **R** | **P** | **R** | **P** |
| **Participants without obesity (N=13)** | | | | | | | | | | | | | | | | |
| Age | 0.159 | 0.605 | 0.147 | 0.632 | 0.257 | 0.397 | 0.197 | 0.519 | 0.298 | 0.322 | 0.367 | 0.217 | 0.308 | 0.306 | 0.347 | 0.246 |
| BW | 0.623 | 0.023 | 0.374 | 0.208 | 0.601 | 0.030 | 0.642 | 0.018 | 0.828 | <0.001 | 0.800 | 0.001 | 0.836 | <0.001 | 0.828 | <0.001 |
| BMI | 0.528 | 0.064 | 0.417 | 0.156 | 0.600 | 0.030 | 0.584 | 0.036 | 0.714 | 0.006 | 0.717 | 0.006 | 0.716 | 0.006 | 0.730 | 0.005 |
| WC | 0.552 | 0.050 | 0.333 | 0.267 | 0.555 | 0.049 | 0.586 | 0.035 | 0.555 | 0.049 | 0.492 | 0.088 | 0.588 | 0.035 | 0.565 | 0.044 |
| HC | 0.519 | 0.069 | 0.134 | 0.662 | 0.491 | 0.089 | 0.520 | 0.068 | 0.537 | 0.059 | 0.497 | 0.084 | 0.548 | 0.053 | 0.530 | 0.063 |
| WHR | 0.335 | 0.263 | 0.388 | 0.190 | 0.364 | 0.222 | 0.386 | 0.193 | 0.309 | 0.305 | 0.254 | 0.403 | 0.348 | 0.244 | 0.329 | 0.272 |
| SBP | -0.131 | 0.670 | -0.067 | 0.828 | -0.180 | 0.557 | -0.144 | 0.639 | 0.101 | 0.744 | 0.089 | 0.771 | 0.052 | 0.866 | 0.055 | 0.858 |
| DBP | 0.452 | 0.121 | 0.221 | 0.468 | 0.510 | 0.075 | 0.477 | 0.099 | 0.589 | 0.034 | 0.633 | 0.020 | 0.445 | 0.128 | 0.520 | 0.069 |
| Glucose | 0.286 | 0.343 | 0.044 | 0.886 | 0.375 | 0.206 | 0.314 | 0.297 | 0.479 | 0.098 | 0.398 | 0.178 | 0.538 | 0.058 | 0.500 | 0.082 |
| Insulin | 0.240 | 0.430 | 0.147 | 0.632 | 0.095 | 0.757 | 0.235 | 0.439 | 0.452 | 0.121 | 0.398 | 0.178 | 0.476 | 0.100 | 0.453 | 0.120 |
| HOMA-IR | 0.257 | 0.397 | 0.114 | 0.712 | 0.142 | 0.643 | 0.257 | 0.397 | 0.466 | 0.109 | 0.404 | 0.171 | 0.497 | 0.084 | 0.471 | 0.105 |
| QUICKI | -0.348 | 0.244 | -0.243 | 0.424 | -0.230 | 0.449 | -0.351 | 0.240 | -0.530 | 0.062 | -0.460 | 0.114 | -0.584 | 0.036 | -0.546 | 0.054 |
| *LEP* mRNA | 0.018 | 0.956 | 0.102 | 0.753 | 0.122 | 0.706 | 0.056 | 0.862 | 0.705 | 0.023 | 0.716 | 0.020 | 0.561 | 0.092 | 0.621 | 0.055 |
| *Adipo* mRNA | -0.193 | 0.571 | -0.170 | 0.616 | -0.178 | 0.601 | -0.221 | 0.515 | -0.274 | 0.444 | -0.302 | 0.397 | -0.156 | 0.666 | -0.215 | 0.551 |
| *Omentin* mRNA | -0.306 | 0.334 | -0.198 | 0.537 | -0.368 | 0.240 | -0.327 | 0.300 | -0.340 | 0.337 | -0.339 | 0.338 | -0.251 | 0.483 | -0.290 | 0.416 |
| *Visfatin* mRNA | 0.056 | 0.863 | 0.010 | 0.974 | 0.117 | 0.716 | 0.039 | 0.903 | -0.095 | 0.794 | -0.138 | 0.704 | 0.029 | 0.936 | -0.036 | 0.922 |
| Se. Leptin | 0.384 | 0.218 | 0.494 | 0.103 | 0.209 | 0.514 | 0.390 | 0.210 | 0.634 | 0.027 | 0.591 | 0.043 | 0.687 | 0.014 | 0.660 | 0.019 |
| Se. Adipo | -0.566 | 0.055 | -0.676 | 0.016 | -0.677 | 0.016 | -0.608 | 0.036 | -0.545 | 0.067 | -0.647 | 0.023 | -0.569 | 0.053 | -0.608 | 0.036 |
| Adipo/Leptin Ratio | -0.614 | 0.044 | -0.644 | 0.033 | -0.526 | 0.097 | -0.626 | 0.039 | -0.607 | 0.048 | -0.636 | 0.035 | -0.709 | 0.015 | -0.693 | 0.018 |
| Se. Omentin | -0.033 | 0.923 | -0.255 | 0.449 | 0.063 | 0.855 | -0.076 | 0.825 | -0.548 | 0.081 | -0.476 | 0.139 | -0.505 | 0.113 | -0.502 | 0.115 |
| Se. Visfatin | -0.082 | 0.810 | -0.053 | 0.878 | 0.049 | 0.887 | -0.062 | 0.856 | 0.033 | 0.924 | 0.111 | 0.745 | 0.007 | 0.984 | 0.047 | 0.891 |
| **Participants with obesity (N=14)** | | | | | | | | | | | | | | | | |
| Age | 0.321 | 0.263 | 0.271 | 0.349 | 0.173 | 0.554 | 0.330 | 0.248 | 0.454 | 0.103 | 0.430 | 0.125 | 0.478 | 0.084 | 0.487 | 0.077 |
| BW | 0.434 | 0.121 | 0.503 | 0.067 | 0.160 | 0.585 | 0.444 | 0.112 | 0.492 | 0.074 | 0.358 | 0.209 | 0.607 | 0.021 | 0.523 | 0.055 |
| BMI | 0.303 | 0.292 | 0.309 | 0.282 | 0.140 | 0.632 | 0.306 | 0.288 | 0.375 | 0.186 | 0.220 | 0.450 | 0.520 | 0.056 | 0.408 | 0.147 |
| WC | 0.319 | 0.266 | 0.304 | 0.291 | 0.186 | 0.523 | 0.317 | 0.270 | 0.455 | 0.102 | 0.275 | 0.342 | 0.575 | 0.031 | 0.464 | 0.094 |
| HC | 0.321 | 0.263 | 0.356 | 0.211 | 0.152 | 0.604 | 0.320 | 0.264 | 0.408 | 0.147 | 0.229 | 0.431 | 0.596 | 0.024 | 0.442 | 0.113 |
| WHR | 0.271 | 0.349 | 0.214 | 0.462 | 0.185 | 0.527 | 0.269 | 0.353 | 0.427 | 0.128 | 0.300 | 0.297 | 0.436 | 0.119 | 0.406 | 0.149 |
| SBP | 0.058 | 0.851 | -0.126 | 0.683 | 0.248 | 0.414 | 0.048 | 0.876 | 0.232 | 0.446 | 0.208 | 0.495 | 0.401 | 0.174 | 0.303 | 0.315 |
| DBP | -0.071 | 0.817 | 0.057 | 0.853 | -0.104 | 0.736 | -0.059 | 0.849 | 0.131 | 0.669 | 0.162 | 0.597 | 0.255 | 0.401 | 0.181 | 0.555 |
| Glucose | -0.002 | 0.995 | -0.250 | 0.388 | 0.290 | 0.315 | 0.017 | 0.954 | 0.062 | 0.833 | 0.019 | 0.949 | 0.230 | 0.430 | 0.131 | 0.656 |
| Insulin | 0.070 | 0.812 | 0.067 | 0.820 | 0.019 | 0.950 | 0.055 | 0.852 | 0.269 | 0.352 | 0.143 | 0.625 | 0.366 | 0.198 | 0.314 | 0.274 |
| HOMA-IR | 0.094 | 0.748 | 0.004 | 0.989 | 0.128 | 0.662 | 0.084 | 0.776 | 0.231 | 0.427 | 0.105 | 0.720 | 0.361 | 0.204 | 0.286 | 0.321 |
| QUICKI | -0.134 | 0.649 | -0.078 | 0.792 | -0.137 | 0.641 | -0.128 | 0.662 | -0.365 | 0.200 | -0.262 | 0.366 | -0.519 | 0.057 | -0.442 | 0.114 |
| *LEP* mRNA | -0.088 | 0.764 | 0.124 | 0.672 | -0.280 | 0.332 | -0.088 | 0.766 | 0.245 | 0.398 | 0.327 | 0.254 | 0.150 | 0.609 | 0.264 | 0.362 |
| *Adipo* mRNA | -0.589 | 0.027 | -0.380 | 0.180 | -0.538 | 0.047 | -0.586 | 0.028 | -0.761 | 0.003 | -0.735 | 0.004 | -0.716 | 0.006 | -0.757 | 0.003 |
| *Omentin* mRNA | -0.511 | 0.074 | -0.591 | 0.033 | -0.214 | 0.483 | -0.512 | 0.074 | -0.513 | 0.088 | -0.448 | 0.144 | -0.464 | 0.129 | -0.494 | 0.103 |
| *Visfatin* mRNA | -0.560 | 0.046 | -0.435 | 0.137 | -0.496 | 0.085 | -0.564 | 0.045 | -0.460 | 0.114 | -0.396 | 0.180 | -0.472 | 0.104 | -0.465 | 0.109 |
| Se. Leptin | 0.322 | 0.262 | 0.249 | 0.390 | 0.211 | 0.469 | 0.293 | 0.309 | 0.364 | 0.201 | 0.247 | 0.394 | 0.482 | 0.081 | 0.409 | 0.147 |
| Se. Adipo | 0.199 | 0.494 | 0.140 | 0.632 | 0.129 | 0.660 | 0.193 | 0.509 | -0.153 | 0.602 | -0.115 | 0.695 | -0.214 | 0.462 | -0.200 | 0.492 |
| Adipo/Leptin Ratio | -0.303 | 0.293 | -0.441 | 0.115 | -0.023 | 0.938 | -0.290 | 0.314 | -0.671 | 0.009 | -0.606 | 0.022 | -0.740 | 0.002 | -0.724 | 0.003 |
| Se. Omentin | -0.475 | 0.086 | -0.407 | 0.149 | -0.418 | 0.137 | -0.516 | 0.059 | -0.361 | 0.204 | -0.351 | 0.218 | -0.499 | 0.069 | -0.449 | 0.108 |
| Se. Visfatin | -0.494 | 0.073 | -0.177 | 0.544 | -0.615 | 0.019 | -0.490 | 0.076 | -0.417 | 0.138 | -0.417 | 0.137 | -0.508 | 0.063 | -0.495 | 0.072 |

N=number; Short-⌀=shortest diameter; Long-⌀=longest diameter; BW=body weight; BMI=body mass index; WC=waist circumference; HC=hip circumference; WHR=waist-to-hip ratio; SBP=systolic blood pressure; DBP=diastolic blood pressure; HOMA-IR=homeostatic model assessment for insulin resistance; QUICKI=quantitative insulin sensitivity check index; Adipo=adiponectin; Se.=Serum; Adipo/Leptin Ratio=serum adiponectin-to-leptin ratio
